# First-Time Detection of *Mycobacterium bovis* in Livestock Tissues and Milk in the West Bank, Palestinian Territories

**DOI:** 10.1371/journal.pntd.0002417

**Published:** 2013-09-12

**Authors:** Suheir Ereqat, Abedelmajeed Nasereddin, Hagai Levine, Kifaya Azmi, Amer Al-Jawabreh, Charles L. Greenblatt, Ziad Abdeen, Gila Kahila Bar-Gal

**Affiliations:** 1 Al-Quds Nutrition and Health Research Institute, Faculty of Medicine, Al-Quds University, Abu-Deis, the West Bank, Palestinian Authority; 2 The Koret School of Veterinary Medicine, The Hebrew University of Jerusalem, Rehovot, Israel; 3 Hebrew University-Hadassah Braun School of Public Health and Community Medicine, Ein Kerem, Jerusalem, Israel; 4 Department of Microbiology and Molecular Genetics, The Hebrew University-Hadassah Medical School, Jerusalem, Israel; Kwame Nkrumah University of Science and Technology (KNUST) School of Medical Sciences, Ghana

## Abstract

**Background:**

Bovine tuberculosis, bTB, is classified by the WHO as one of the seven neglected zoonontic diseases that cause animal health problems and has high potential to infect humans. In the West Bank, bTB was not studied among animals and the prevalence of human tuberculosis caused by *M. bovis* is unknown. Therefore, the aim of this study was to estimate the prevalence of bTB among cattle and goats and identify the molecular characteristics of bTB in our area.

**Methodology/principal findings:**

A total of 208 tissue samples, representing 104 animals, and 150 raw milk samples, obtained from cows and goats were examined for the presence of mycobacteria. The tissue samples were collected during routine meat inspection from the Jericho abattoir. DNA was extracted from all samples, milk and tissue biopsies (n = 358), and screened for presence of TB DNA by amplifying a 123-bp segment of the insertion sequence IS*6110*. Eight out of 254 animals (3.1%) were found to be TB positive based on the IS*6110*-PCR. Identification of *M. bovis* among the positive TB samples was carried out via real time PCR followed by high resolution melt curve analysis, targeting the A/G transition along the *oxyR* gene. Spoligotyping analysis revealed a new genotype of *M. bovis* that was revealed from one tissue sample.

**Significance:**

Detection of *M. bovis* in tissue and milk of livestock suggests that apparently healthy cattle and goats are a potential source of infection of bTB and may pose a risk to public health. Hence, appropriate measures including meat inspection at abattoirs in the region are required together with promotion of a health campaign emphasizing the importance of drinking pasteurized milk. In addition, further studies are essential at the farm level to determine the exact prevalence of bTB in goats and cattle herds in the West Bank and Israel.

## Author Summary

Bovine tuberculosis (caused by *Mycobacterium bovis*) is a zoonotic disease causing major public health concerns. Animals, both wild and domestic, tested positive to bovine tuberculosis are being slaughtered and the affected herds are placed under temporary movement restrictions. Hence, bTB can have strong economic implications. In the West Bank, bTB has not been reported during the past 20 years. Our study determined the presence of *M. bovis* in tissue and milk samples of goats and cattle. Tissues were collected from slaughtered animals during routine meat inspection at the abattoir and were categorized as negative for mycobacteria. The frequency of the detected bTB was low but indicated the low sensitivity of the routine meat inspection compared to diagnosis of *M. bovis* infection via PCR assay. Furthermore, *M. bovis* was found in goat milk samples. As bTB is considered a neglected disease, known in high frequency in Africa and Asia, we recommend conducting a regional survey to assess the prevalence of the disease among cattle, sheep and goat herds. The results of the survey will undoubtedly be a platform for new public health policies.

## Introduction

Tuberculosis (TB) is included among the neglected diseases that disproportionally affect the world low-income populations. Bovine tuberculosis (bTB) is a zoonotic disease causing major public health concerns. It is caused by *Mycobacterium bovis*, a member of the *Mycobacterium tuberculosis* complex (MTC). The various ecotypes of *M. bovis* have a wide host range and can affect different target species, including domesticated and wild animals, mainly cattle [1], [2]. The global prevalence of human TB caused by *M. bovis* was estimated to be 3.1% of all human TB cases worldwide, accounting for 2.1% and 9.4% of pulmonary and extra pulmonary TB cases, respectively [3]. Several studies, especially in developing countries, have reported the presence of mycobacteria among slaughtered animals. In Nigeria and Ethiopia, bTB was identified among slaughtered goats with a prevalence of 4.5% and 4.2% respectively [4], [5]. In Pakistan, a 2.4% prevalence of bTB was reported in goat herds based on the tuberculin test [6]. In Egypt, bTB was highly prevalent in cattle and buffalos during the 1980s and ranged between 6.9%–26.2%. Control programs reduced the presence of the disease to 2.6% during the 1990's and the latest survey, conducted in seven governorates in Egypt, indicated that the prevalence had been reduced to 0.05% [7]. In the neighboring countries of Jordan and Lebanon, the disease was detected and reported in animals with no formal statistics [8]. Prior to this study, the only confirmed bTB cases in Israel and the West Bank were in 1990 at four dairy farms at the Golan Heights (n = 386). Due to effective test-and-slaughter policy, the disease was eradicated [9], and since then, there have been no reports of bTB. In the West Bank and in Israel the cattle herds are not vaccinated against bTB. In Israel the calves are checked by the intradermal tuberculin test (ITT) and positive cases are eradicated (http://www.vetserv.moag.gov.il/Vet/Yechidot/VetBasade/). Due to the low prevalence of the disease among animals and its absence in human TB cases, the routine surveillance for bTB has been neglected, unlike other zoonotic diseases with public health concern such as brucellosis, rabies and leishmaniasis [10], [11].

Cattle, being a major source of meat and milk, play an important role in the economic and social life worldwide, especially in developing countries of Africa and Asia [12]. bTB is widely distributed in the developing countries, since the control measures are not applied and pasteurization is rarely practiced [13]. Aerosol exposure to *M. bovis* is considered to be the most frequent route of infection between cattle and other animals, when sharing the same pasture and the same shelter [4], [5]. Ingestion of infected food such as raw milk, milk products and under cooked meat is considered the primary route of infection between cattle and humans [14]. Generally, to prevent TB infection, farmers are vaccinating their herds using bacillus Calmette-Guerin (BCG), which has been shown to have variable efficacy in cattle, as in humans [15]. In addition, TB elimination programs in domesticated herds together with milk pasteurization have successfully reduced the incidence rate of TB caused by *M. bovis* among cattle and humans alike in developed countries [16].

The prevalence of TB infection in animals has usually been determined by the intradermal tuberculin test (ITT). Recently, it was demonstrated that the ITT has limitations especially misidentification of infected specimens [17]. Hence, polymerase chain reaction (PCR) based methods have the potential of being the most efficient test for detection and identification of *M. bovis* [17]. In this regard, different gene targets have been used to detect and identify *M. bovis* in different clinical samples [16], [18]. To the best of our knowledge, no studies concerning bTB have previously been conducted in the West Bank. The goals of this study were to estimate the prevalence of bTB among cattle and goats in the West Bank and identify the molecular characteristics of the bTB in our region.

## Materials and Methods

### Animal population and sample collection

In the West bank, cattle (*Bos tarus*) and goats (*Capra hircus*) are generally raised together at small (10–50 heads) or intermediate (51–100 heads) unit farms. Most of them are dairy and/or beef farms. The goats are raised mainly as pastoral herds with food supplements given every day at the enclosure. The cattle are managed under an intensive feeding system at the farm. The most abundant goat breeds are local Baladi and Shamit breeds. The cattle are mainly of dairy breeds, Friesians breed, where females are raised and milked at least twice a day and males are slaughtered for meat. Both species are well adapted to the environment and the climate in the West Bank and Israel.

#### (i) Raw milk samples

**Figure 1:**
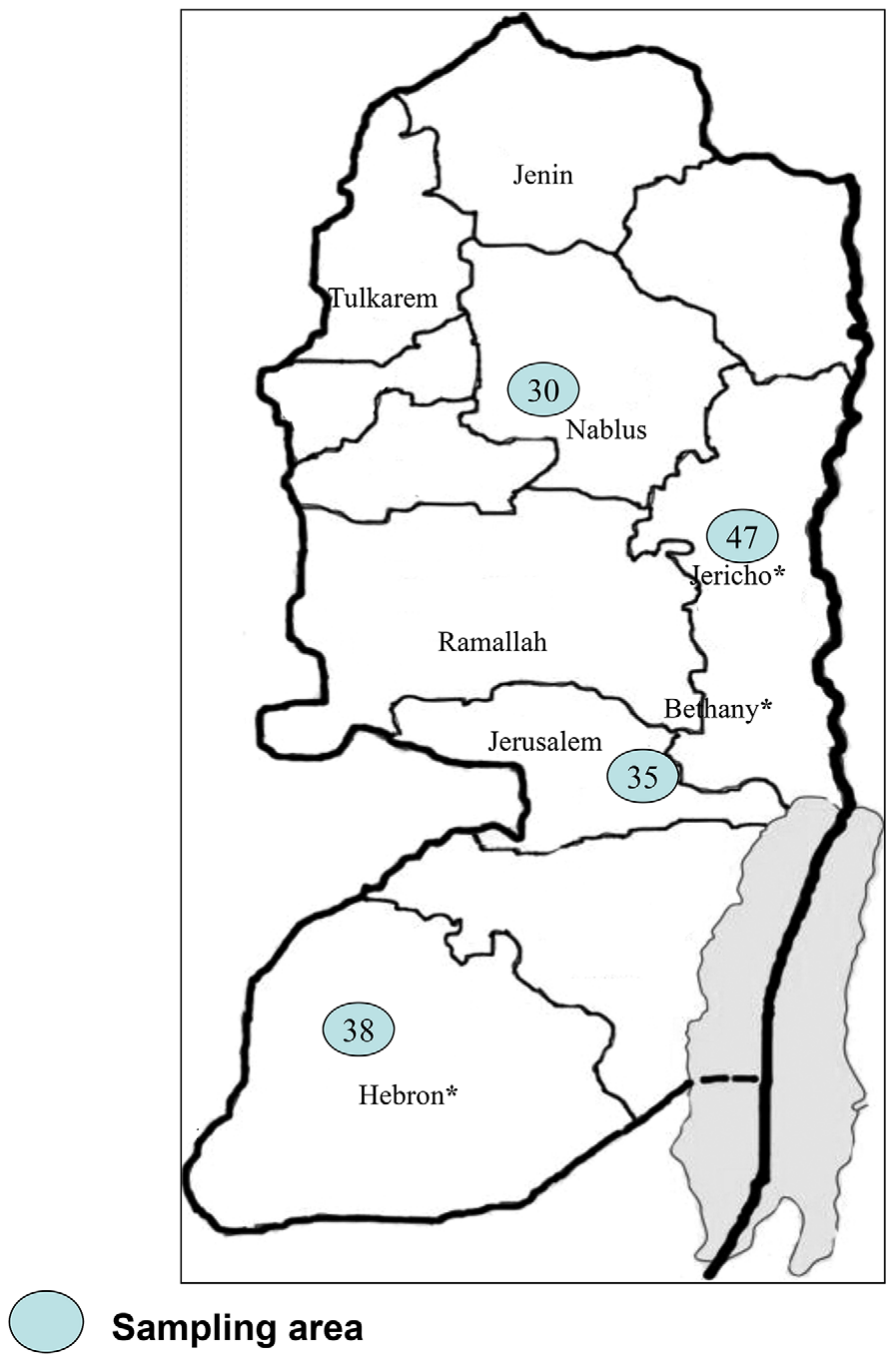
Distribution of samples collected in the West Bank, Palestinian Territories. Numbers indicate the milk samples per district. Asterisk indicates positive *M. bovis* identified in milk samples.

A total of 150 milk samples obtained from 120 goats and 30 cows were collected and tested for bTB. The goat milk samples were collected during 2009 from small farms located in three different areas in the West Bank (Jericho, Hebron and Bethany) which were chosen randomly (Fig. 1). The cow milk samples were collected from one farm in the Nablus district during 2010. This farm is considered to be the distributor farm where most farmers in the West Bank purchase their cattle (Fig. 1).

Prior to collecting the milk the teats were thoroughly cleaned with alcohol to avoid environmental contamination. A composite milk sample (30–40 ml) was collected in a sterile 50 ml tube by hand milking, discarding the first 10–20 ml. All samples were transported refrigerated, and processed within 24 hours after collection. All owners gave their written informed consent to have their animals milked.

#### (ii) Tissue biopsies

A total of 104 animals [44 male goats and 60 cattle (6 females and 54 males)] were tested for bovine TB. From each animal a mesenteric and a lung lymph node tissue biopsy was sampled, for a total of 208 tissue samples. All tissue samples were collected from animals slaughtered at the Jericho abattoir (30 Kilometers east of Jerusalem) during routine meat inspection. Routine meat inspection was conducted by the local meat inspector according to procedures recommended by the Palestinian ministry of agriculture. It involved postmortem visualization of pathological lesions in lung, liver and kidneys. All the examined animals were found to be healthy, with no signs of infection. Thus, samples were collected from all the animals (goats and cattle), which were slaughtered, between late October and December 1 2011. Each tissue sample was divided into two pieces, one was kept frozen for further analysis and the other was used in the current study. The origin of the cattle could not be traced due to poor documentation and multiple sales of the animals before slaughter. Most of the calves slaughtered in the Jericho abattoir were purchased from distributors in the Palestinian districts such as Nablus and Hebron. These distributors are known to buy calves from Israeli farmers. On the other hand, goats slaughtered in the Jericho abattoir were purchased from local Jericho farmers and originated from different Palestinian districts. The age of the animals ranged from 2 to 5 years.

### Smear preparation and tissue culture

Approximately 2 g of tissue from each sample (n = 208) was cut into small pieces with a sterile scalpel blade and homogenized with 1.0 ml of sterile distilled water using a pestle and a mortar. Two loops full of tissue homogenate were smeared on glass slides. The smears were dried, heat fixed, stained with Ziehl-Neelsen (ZN) and examined for Acid Fast Bacilli (AFB). In an attempt to isolate *M. bovis*, the tissue homogenates (200 µl each) that were found TB positive by PCR were decontaminated with 4% NaOH [19], inoculated onto two slants of Lowenstein-Jensen (LJ) media with and without glycerol and incubated for 6–8 weeks at 37°C.

### DNA extraction

Ten milliliters of milk samples were centrifuged at 13000 rpm for 15 min. A sterile cotton swab was used to remove the fat layer, and the supernatant was discarded. The pellet was re-suspended in 200 µl of tissue lysis buffer. DNA extraction was completed following the manufacturer's guidelines (Master Pure DNA Purification kit, Ambion, MG7110, Madison WI, USA). DNA was extracted from the tissue samples via the Qiagen DNA extraction kit (QIAGEN GmbH, 40724 Hilden, Germany). The eluted DNA (100 µl) was washed and concentrated by Amicon Ultra-0.5 ml filters (Millipore corporation, Billerica, MA 01821).

### Detection of MTB DNA by IS*6110*-PCR

All DNA extracts from both milk and tissue biopsies (n = 358) were screened for the presence of TB by amplifying a 123-bp segment of the insertion sequence *IS6110* [20]. Two microliters from the extracted DNA were used for amplification. The PCR was conducted using Taq DNA polymerase and accompanying reagents (QIAGEN GmbH, Hilden, Germany). The specificity of the amplified target (123 bp) was confirmed by restriction fragment length polymorphism (RFLP) analysis using *HaeIII* enzyme [21]. The samples that were TB positive were further analyzed to determine the specific mycobacterium strain.

### Identification of *M. bovis* by real time PCR- high resolution melt curve analysis

Our laboratory has described the use of real time PCR with high resolution melt curve analysis (rPCR-HRM) to differentiate between *M. bovis* and *M. tuberculosis* in human clinical samples [22]. Here, we used the rPCR-HRM assay to directly identify *M. bovis* in clinical samples of bovine origin. Assuming that *M. bovis* is often found at low concentrations in bovine specimens, a fragment of 200 bp, targeting an A/G transition of the *oxyR* gene, was amplified using previously published primers (LC90 and LC91) [23], then PCR product (1 µl) was subjected to the real-time PCR followed by HRM analysis as described in our previous study [22]. Briefly, each reaction mixture contained: 10 µl of 2× Thermo-start PCR Master Mix (Thermo scientific), 1.5 µM SYTO 9 (Invitrogen), primer mixes were used at 250 µM final concentrations. Purified DNA from three human clinical isolates, previously identified as MTB, and from the reference strains (*M. tuberculosis* (H37Rv) and *M. bovis* BCG) were included as positive controls in all PCR amplifications conducted on the samples. DNA from the clinical samples and controls were added in 2 µl volumes in a total reaction of 20 µl. The PCR was as follows: hold at 95°C for 15 min for a hot start reaction, then 40 cycles of amplification with 5 s denaturation, 10 s annealing and extension at 54°C with a temperature rise of 0.2°C at each step. The real time PCR-HRM products were subjected to RFLP-based analysis using the *AluI* restriction enzyme. The DNA fragments were analyzed on 3% agarose gel and visualized under UV light. The expected sizes of the digested amplicons were 139 bp and 12 bp for *M. tuberculosis*, and 79 bp, 60 bp and 12 bp for *M. bovis*. Since the 12 bp is too small to visualize, we optimized the gel electrophoresis to visualize one band for *M. tuberculosis* (139 bp) and two bands for *M. bovis* (79 bp and 60 bp). To confirm the presence of typical *M. bovis* in the DNA extracts we targeted the RD4 region. Presence or absence of this region allows distinction between *M. bovis* and *M. caprae* [24].

### Genotyping

Spoligotyping analysis of the positive amplification samples together with known representative genotypes (positive controls) were performed as described previously [25]. The positive controls included the reference strain, *M. tuberculosis* (H37Rv) and *M. bovis* BCG.

## Results

### Detection and identification of MTB DNA in milk and tissue samples

**Figure 2:**
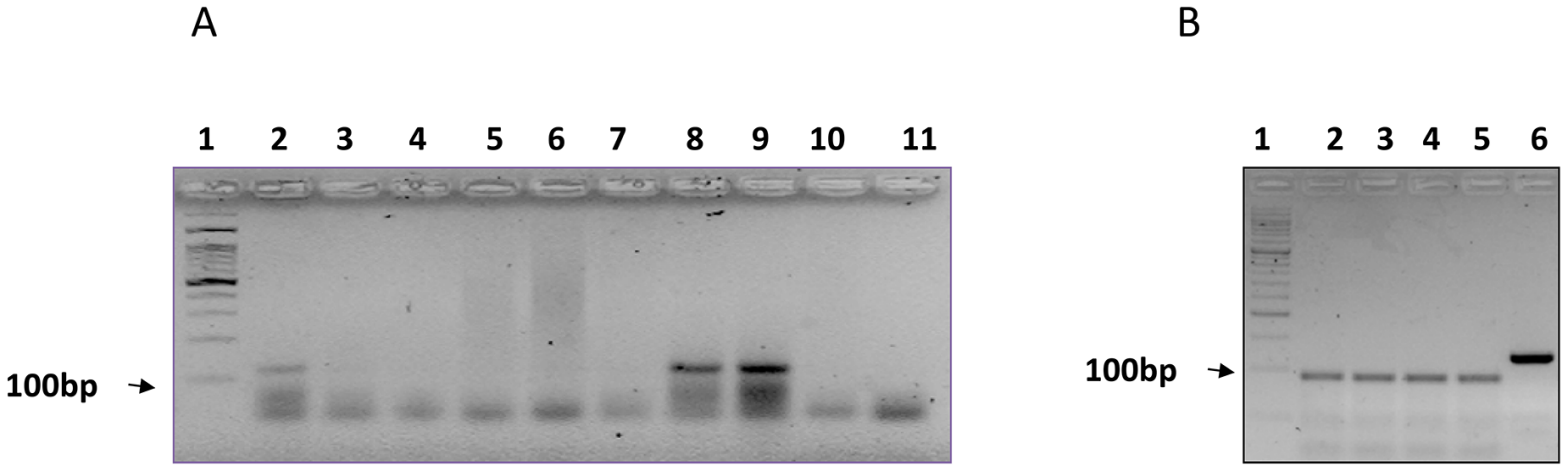
Detection of TB among animals in the West Bank. **A**) Positive amplification of *IS6110* gene fragment (123 bp) among DNA extracts from milk samples. Lane 1 = l00 bp ladder, Lanes 2, 8, 9 = positive amplification of milk samples, Lane 10 = Blank PCR control, Lane 11 = Blank extraction control. **B**) Confirmation of MTB complex by restriction enzyme *HaeIII*. Lane 1 = 50 bp ladder, Lanes 2–5 = samples were digested indicating the presence of MTB complex; Lane 6 = undigested sample (H37Rv).

Attempts to amplify the *IS6110*-PCR region from all DNA extracts, milk and tissue biopsies were carried out. A sample was considered TB positive when a band of 123 bp was observed on a three percent garose gel (Fig. 2A). Altogether ten DNA extracts were successfully amplified indicating the presence of TB in both milk and tissue biopsy. Negative controls (blank extraction and blank PCR) were always clean during the PCR amplification assay supporting the authenticity of the results. Among the milk samples, four samples originating from goats were TB positive (4/150, 2.7%): two from Bethany, one from Hebron and one from Jericho (Fig. 1; Table 1). All milk samples derived from cows (n = 30) were negative for TB (Table 1). Among the analyzed tissue samples, six samples (6/208, 2.9%) obtained from four animals (3 calves and 1 goat) were found TB positive by IS*6110*-PCR (Table 2). In two calves (2 and 3 years old), TB DNA was amplified from both the lung and mesenteric lymph nodes. In the other two animals (2 and 2.5 years old), TB was identified only in the mesenteric lymph node (Table 2). The specificity of the amplified IS*6110* gene target (123 bp) was confirmed by RFLP using *HaeIII* enzyme. The expected 94 bp and 29 bp fragments were observed in all positive samples (Fig. 2B). Altogether, eight out of 254 animals (3.1%) were found TB positive, of which four were goat milk samples, and four (three calves and one goat) were tissue biopsies sampled at the Jericho abattoir. All the three calves that were found to be TB positive were bought from Israeli farmers.

**Table 1:** Detection of MTB DNA in milk samples based on amplification of *IS6110* gene.

Species	Total number of animals tested	PCR positive (%)
Goat	120	4 (2.7)
Cow	30	0
Total	150	4 (2.7)

**Table 2:** Detection of MTB DNA in tissue biopsy samples based on amplification of *IS6110* gene.

PCR positive				
Species	Total number of animals tested	Mesenteric lymph node	Lung lymph node and Mesenteric lymph node	Total (%)
Goat	44	1	0	1 (2.3)
Cow/calf	60	1	2	3 (5.0)
Total	104	2	2	4 (3.8)

### Identification of *M. bovis* in milk and tissue samples by real-time PCR-HRM

**Figure 3:**
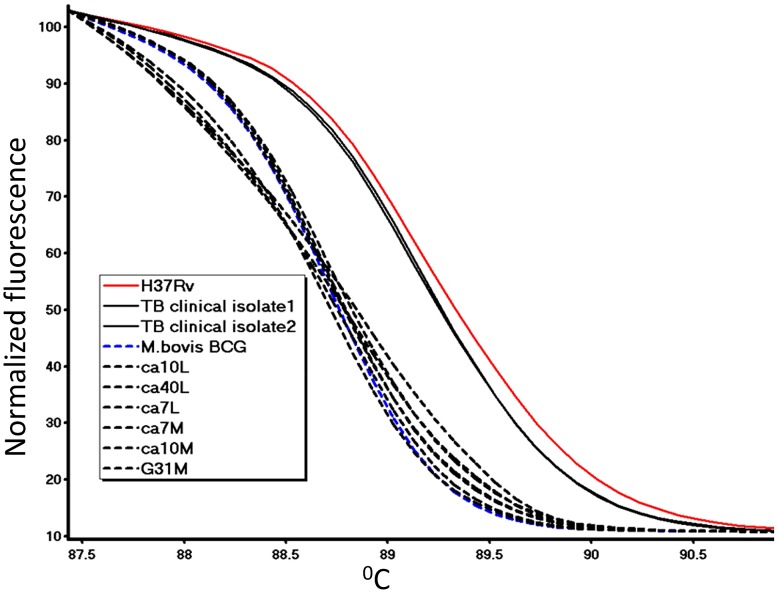
Normalized melting curves for studied animal clinical samples and TB clinical isolates. Dashed lines represent *M. bovis* DNA; solid lines represent MTB DNA. All reactions were performed in duplicate. Positive controls are H37Rv and BCG.

All ten samples found TB positive by IS*6110*-PCR were further studied to identify the TB strain based on the SNP of the *oxyR* gene. As shown by the normalized melt curves (Fig. 3), the real-time PCR-HRM assay distinguishes between MTB isolates and *M. bovis*. All positive animal samples (milk and tissue) were identified as *M. bovis* (Fig. 3).

**Figure 4:**
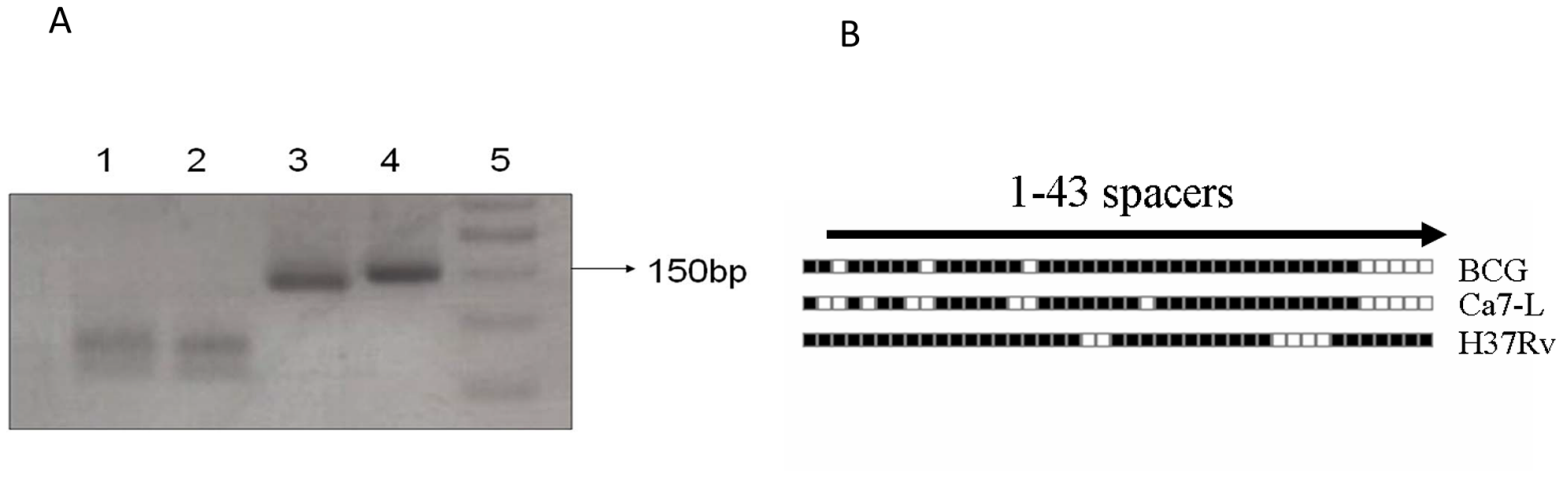
Identification of *M.* *bovis* in the studied samples: A) Identification of *M. bovis* by PCR-RFLP analysis of *oxyR* gene. Lanes 1and 2: *M. bovis* detected in milk samples (showing the 2 bands pattern); Lane 3: positive control (H37Rv; showing one band pattern); Lane 4: undigested sample; Lane 5: 50 bp DNA ladder. **B**) Spoligotyping pattern of *M. bovis* detected in lung tissue of cattle (Ca-7L). The reference strains (*M. bovis* BCG and H37Rv) were included as positive control**s**.

The results of the real-time PCR HRM assay were also confirmed by *Alu I* enzyme digestion, which showed the two expected bands pattern of *M. bovis* (79 bp and 60 bp) (Fig. 4 A). Moreover, all positive tissue samples were confirmed as *M. bovis* based on the RD4 deletion typing while all milk samples (n = 4) were negative, probably due to paucibacillary of the milk extracts. For one tissue sample obtained from a calf (ID: ca7-L), identification of *M. bovis* was also confirmed by direct sequencing of the *oxyR* gene fragment. The sequence was found to be 100% identical to the published *M. bovis* sequence (BX248342.1) and was deposited to the gene bank (Accession no. JQ866621).

### Genotyping

Direct spoligotyping of nine DNA extracts failed to give a full pattern and no type could be determined despite the fact that all of them were positive by *IS6110*-PCR. One sample (ID:ca7-L) gave a full spoligotype pattern and revealed a new genotype of *M. bovis* according to the spoligotype database website (www.mbovis.org) (Fig. 4 B).

### Microscopic examination and culture results

**Figure 5:**
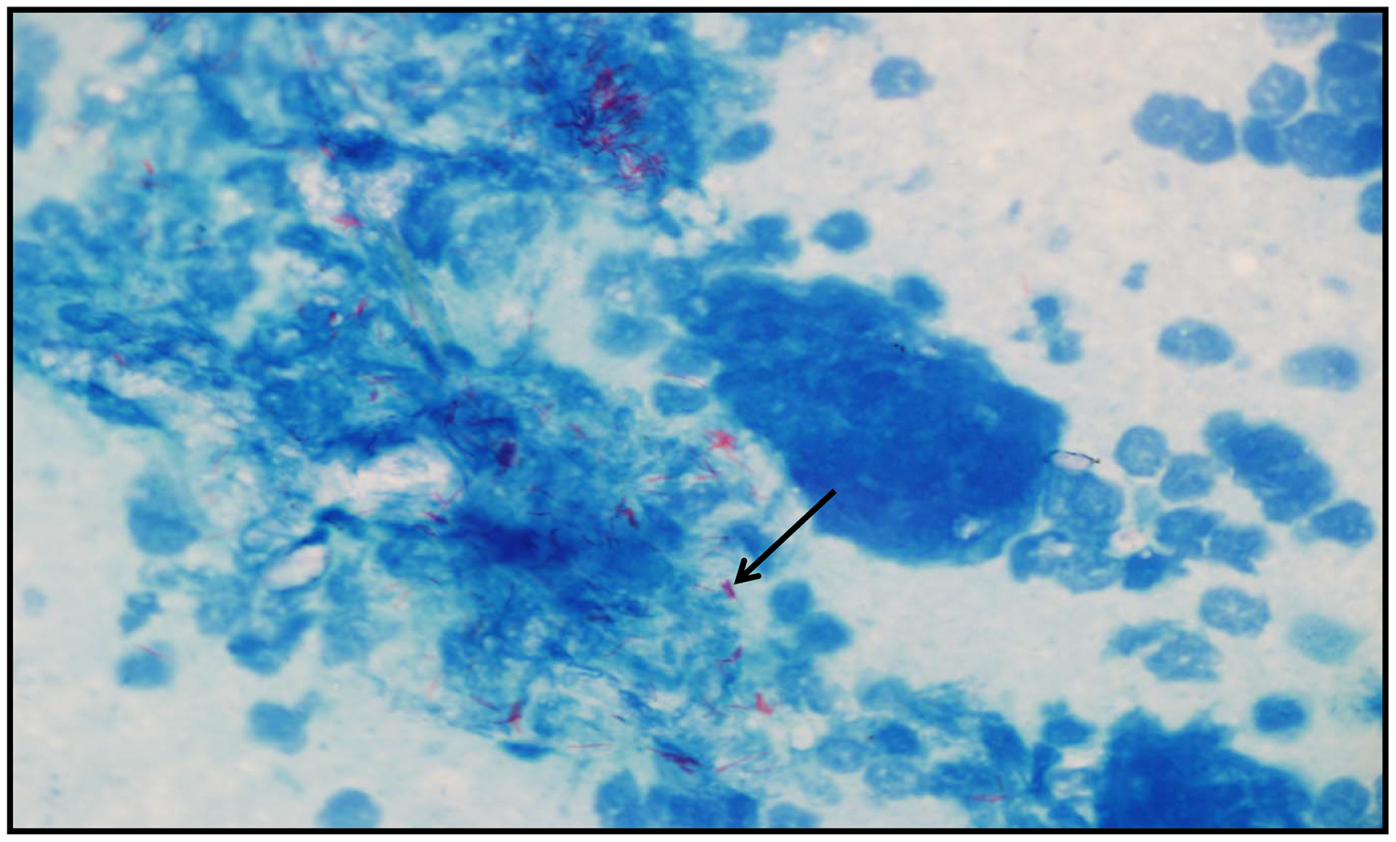
ZN- stained smear obtained from a cattle lung lymph node (Ca-7L), showing the presence of Acid Fast Bacilli (×1000).

Among the 208 tissue smears that were tested, AFBs were detected in only one sample (ID:ca-7L)(Fig. 5). No bacterial growth was observed from all cultured tissue samples after 8 weeks of incubation.

## Discussion

Identification of *M. bovis* in raw milk and tissue samples of domestic calves and goats from the West Bank is an important finding with economic and public health consequences. In developed countries, bTB has been almost eradicated after implementing prevention and control measures [26], [27] but due to globalization and intensive trade of livestock, bTB has become a risk again. Animals purchased from a high bTB incidence area and introduced into a low bTB incidence region increase the risk of a herd breakdown as found in England and Ireland [26], [28], [29]. The prevalence of human tuberculosis caused by bTB is unknown. Even in developed countries the HIV pandemic has raised concerns about transmission of *M. bovis* to and between human individuals. Therefore, eradication of bTB is important for humans, livestock and the wildlife population worldwide. To the best of our knowledge, no cases of bTB have been reported within the last two decades in the West Bank. The finding of bTB in three calves that were purchased from Israeli farmers and slaughtered in the Jericho abattoir strengthens the importance of joint regional activities to prevent an outbreak. Israel and the Palestinian territories are considered one geographical region. The close contact between Israeli and Palestinian farmers can be a route for transmission of bTB. Furthermore, this geographical region serves as a bridge between Africa, Asia and Europe and a route for transmission of various infectious diseases including zoonotic diseases. International and local trade together with movement of cattle, sheep and goats within and between Israel, Palestinian territories, Egypt, Jordan and Lebanon are considered the main factors for the transmission of the disease. Therefore, management of the disease can be achieved only with regional planning, including enforcement and implementation of the trading laws for animals [26].

The results of the current study showed that positive identification of bTB could be obtained from three different sources (milk, lung and mesenteric lymph nodes) using comprehensive methodologies. Because the milk and tissue biopsy samples were obtained from different individuals it is not possible to conclude which sample/tissue should be chosen for examination in the first place. Detection of *M. bovis* in lung and mesenteric lymph node may be associated with the route of infection and the close contact between animals facilitates the spread of tuberculosis [30]. The failure (with one exception) to spoligotype the bTB positive milk samples and tissue extracts may be related to the low bacterial concentrations and/or uneven distribution within a single lymph node [31]. The culture of *M. bovis* remains the gold standard for definitive diagnosis of bTB. Unfortunately, in spite of all our attempts no bacterial growth was observed in all cultured tissue samples, after 8 weeks of incubation, possibly due to loss of pathogen viability caused by improper storage and/or a delay in sample processing.

Definitive diagnosis of TB in live animals and especially identification of the specific pathogen is important knowledge for veterinary and public health management. The low number of bacteria and the low sensitivity of the intradermal tuberculin test, which failed to detect the infected cattle during an outbreak [17], can impede the detection rates and further stresses the importance of using a PCR-based assay for bTB detection. The drawback of the high sensitivity of the PCR assay is possible contamination that might result in a false positive diagnosis of bTB. In our study we carried out all the precautions to avoid cross contamination between samples, including separated areas for extraction, PCR set-up and post-PCR analysis. Moreover, the specificity of the findings obtained by *IS6110*-PCR was confirmed using other molecular biology techniques (RFLP analysis, direct sequencing and deletion typing). In all the PCR assays the blank controls were negative, supporting the authenticity of the results.

It is noteworthy that all animals included in this study were apparently healthy and did not show any signs of infection. This may reflect the ability of *M. bovis* to survive in the host at a latent state, as development of clinical signs in livestock may take years [32]. The lack of AFB in all but one of the tested tissue smears further supports the hypothesis that animals may be in the early stages of infection; since pathogens can be visualized and detected once they overcome a critical limited quantity (at least 5×10^4^ mycobacteria/ml) of organisms present in the sample [33]. One sample (lung lymph node) was identified as TB positive based on the microscopic examination. The lack of lung lesions was unexpected considering the heavy bacterial load that was observed by microscopic examination, which usually occurs in advanced stages of the disease. However, *M. bovis* is more likely to be extra pulmonary [34] and the presence or absence of visible lesions may not result in a correct visual examination, since isolation of *M. bovis* has been confirmed from non visible lesions [30]. Consequently, in the abattoirs there is a requirement for additional diagnostic methods, more sensitive than the pathology, to identify infected animals. The presence of *M. bovis*, in slaughtered animal meat is considered a health risk for the abattoir workers [16]. Thus, establishment of facilities that fit standard requirements with occupational health and safety measures are required to protect the health of abattoir and farm workers in our region. Recently, the Palestinian Ministry of Health has regulated the slaughtering of animals (cattle, goats as well as poultry) in designated abattoirs instead of what was commonly practiced (near butcher shops and local houses) throughout the West Bank.

This study shows that we can apply the real-time PCR HRM assay to examine raw milk and tissue biopsies for the presence/absence of *M. bovis*. Screening the livestock on a regular basis will prevent transmission of *M. bovis* to other herds and humans and *vice versa* [35]. Spoligotyping revealed a new genotype of *M. bovis* in one tissue sample (ID:ca7-L). Such an approach will help us to identify the predominant genotypes and compare them with those circulating in neighboring countries. This will indicate whether some genotypes have an advantage over others and are increasing among animals in our region.

In conclusion, detection of *M. bovis* in tissue and milk of livestock in the West Bank suggests that asymptomatic and apparently healthy cattle and goats are a potential source of infection of bTB to all those who come into contact with these animals including owners and their families, herdsmen, slaughterhouse workers, abattoir workers, dealers, veterinarians and even pets. Currently, the source of the disease and its route into the region is unknown. Further laboratory testing, periodic sampling from different abattoirs and additional epidemiological studies at the herd level should be conducted to determine the exact bTB prevalence and to further understand the mode of transmission. The role of wildlife in transmission of the *M. bovis* is unknown although theoretically it can represent potential reservoirs like the badgers [36]. We believe that in addition to the domestic animals, the wildlife should be studied, as wild species' movements including border crossings can be also a risk factor.

In the West Bank and particularly in rural and Bedouin areas around Jericho and Hebron most cattle herds are raised according to known tradition which includes consumption of raw milk and raw meat, coming in close contact with cattle and goats and even sleeping in and around animal sheds. Furthermore, dairy products are habitually produced, stored and transported under insufficient hygienic standards and considered risk factors for spreading and infecting humans throughout the Palestinian Territories with bTB. Therefore, it is essential to launch health awareness campaigns in the West Bank and in particular in the rural and Bedouin areas in the region.

A national and regional bTB survey is a priority in Palestinian Territories and Israel due to a total absence of national figures due to the assumption that bTB was eradicated in 1990. Such a survey will undoubtedly be a platform for new public health policies.

## References

[1] Medeiros L, Marassi CD, Duarte RS, da Silva MG, Lilenbaum W (2011) Comparison of decontamination methods for primary isolation of Mycobacterium bovis in paucibacillary bovine tissues. Lett Appl Microbiol 54: 182–186. 22118726 10.1111/j.1472-765X.2011.03185.x

[2] Smith RM, Drobniewski F, Gibson A, Montague JD, Logan MN, et al (2004) Mycobacterium bovis infection, United Kingdom. Emerg Infect Dis 10: 539–541. 15109433 10.3201/eid1003.020819 PMC3322792

[3] Ayele WY, Neill SD, Zinsstag J, Weiss MG, Pavlik I (2004) Bovine tuberculosis: an old disease but a new threat to Africa. Int J Tuberc Lung Dis 8: 924–937. 15305473

[4] Cadmus SI, Adesokan HK, Jenkins AO, van Soolingen D (2009) Mycobacterium bovis and M. tuberculosis in goats, Nigeria. Emerg Infect Dis 15: 2066–2067. 19961707 10.3201/eid1512.090319 PMC3044523

[5] Hiko A, Agga GE (2010) First-time detection of mycobacterium species from goats in Ethiopia. Trop Anim Health Prod 43: 133–139. 20725858 10.1007/s11250-010-9665-4

[6] Javed MT, Munir A, Shahid M, Severi G, Irfan M, et al (2010) Percentage of reactor animals to single comparative cervical intradermal tuberculin (SCCIT) in small ruminants in Punjab Pakistan. Acta Trop 113: 88–91. 19732736 10.1016/j.actatropica.2009.08.026

[7] WHO (1994) Zoonotic tuberculosis (Mycobacterium bovis): memorandum from a WHO meeting (with the participation of FAO). Bull World Health Organ 72: 851–857. 7867130 PMC2486730

[8] OIE. 10th Conference of the OIE Regional Commission for the Middle East; 2009; Doha (Qatar).

[9] Shimshony A (1992) Veterinary public health in Israel. Rev Sci Tech 11: 77–98. 1525425 10.20506/rst.11.1.596

[10] David D, Dveres N, Davidson I, Yagil J, Dvorkin Z, et al (2012) Geographic Translocation of Dog Rabies by Tourism. Israel Journal of Veterinary Medicine 67: 139–141.

[11] Jaffe CL, Baneth G, Abdeen ZA, Schlein Y, Warburg A (2004) Leishmaniasis in Israel and the Palestinian Authority. Trends Parasitol 20: 328–332. 15193564 10.1016/j.pt.2004.05.001

[12] Zinsstag J, Schelling E, Roth F, Kazwala R (2006) Mycobacterium bovis in Africa. In: Thoen CO, Steele JH, Gilsdorf MJ, editors. Mycobacterium bovis infection in animals and humans. 2^nd^ ed. Ames: Iowa State University press. pp. 199–210.

[13] Bakshi CS, Shah DH, Verma R, Singh RK, Malik M (2005) Rapid differentiation of Mycobacterium bovis and Mycobacterium tuberculosis based on a 12.7-kb fragment by a single tube multiplex-PCR. Vet Microbiol 109: 211–216. 16005166 10.1016/j.vetmic.2005.05.015

[14] Biet F, Boschiroli ML, Thorel MF, Guilloteau LA (2005) Zoonotic aspects of Mycobacterium bovis and Mycobacterium avium-intracellulare complex (MAC). Vet Res 36: 411–436. 15845232 10.1051/vetres:2005001

[15] Buddle BM, Keen D, Thomson A, Jowett G, McCarthy AR, et al (1995) Protection of cattle from bovine tuberculosis by vaccination with BCG by the respiratory or subcutaneous route, but not by vaccination with killed Mycobacterium vaccae. Res Vet Sci 59: 10–16. 8525078 10.1016/0034-5288(95)90023-3

[16] Taylor GM, Worth DR, Palmer S, Jahans K, Hewinson RG (2007) Rapid detection of Mycobacterium bovis DNA in cattle lymph nodes with visible lesions using PCR. BMC Vet Res 3: 12. 17567891 10.1186/1746-6148-3-12 PMC1904440

[17] Zarden CF, Marassi CD, Figueiredo EE, Lilenbaum W (2013) Mycobacterium bovis detection from milk of negative skin test cows. Vet Rec 172: 130. 10.1136/vr.101054 PMC3582086 23292843

[18] Vitale F, Capra G, Maxia L, Reale S, Vesco G, et al (1998) Detection of Mycobacterium tuberculosis complex in cattle by PCR using milk, lymph node aspirates, and nasal swabs. J Clin Microbiol 36: 1050–1055. 9542936 10.1128/jcm.36.4.1050-1055.1998 PMC104688

[19] Rahim Z, Mollers M, te Koppele-Vije A, de Beer J, Zaman K, et al (2007) Characterization of Mycobacterium africanum subtype I among cows in a dairy farm in Bangladesh using spoligotyping. Southeast Asian J Trop Med Public Health 38: 706–713. 17883011

[20] Eisenach KD, Cave MD, Bates JH, Crawford JT (1990) Polymerase chain reaction amplification of a repetitive DNA sequence specific for Mycobacterium tuberculosis. J Infect Dis 161: 977–981. 2109022 10.1093/infdis/161.5.977

[21] Zink A, Haas CJ, Reischl U, Szeimies U, Nerlich AG (2001) Molecular analysis of skeletal tuberculosis in an ancient Egyptian population. J Med Microbiol 50: 355–366. 11289521 10.1099/0022-1317-50-4-355

[22] Ereqat S, Bar-Gal GK, Nasereddin A, Azmi K, Qaddomi SE, et al (2010) Rapid differentiation of Mycobacterium tuberculosis and M. bovis by high-resolution melt curve analysis. J Clin Microbiol 48: 4269–4272. 20844219 10.1128/JCM.00943-10 PMC3020821

[23] Stermann M, Bohrssen A, Diephaus C, Maass S, Bange FC (2003) Polymorphic nucleotide within the promoter of nitrate reductase (NarGHJI) is specific for Mycobacterium tuberculosis. J Clin Microbiol 41: 3252–3259. 12843072 10.1128/JCM.41.7.3252-3259.2003 PMC165301

[24] Pinsky BA, Banaei N (2008) Multiplex real-time PCR assay for rapid identification of Mycobacterium tuberculosis complex members to the species level. J Clin Microbiol 46: 2241–2246. 18508937 10.1128/JCM.00347-08 PMC2446918

[25] Kamerbeek J, Schouls L, Kolk A, van Agterveld M, van Soolingen D, et al (1997) Simultaneous detection and strain differentiation of Mycobacterium tuberculosis for diagnosis and epidemiology. J Clin Microbiol 35: 907–914. 9157152 10.1128/jcm.35.4.907-914.1997 PMC229700

[26] Humblet MF, Boschiroli ML, Saegerman C (2009) Classification of worldwide bovine tuberculosis risk factors in cattle: a stratified approach. Vet Res 40: 50. 19497258 10.1051/vetres/2009033 PMC2710499

[27] Etter E, Donado P, Jori F, Caron A, Goutard F, et al (2006) Risk analysis and bovine tuberculosis, a re-emerging zoonosis. Ann N Y Acad Sci 1081: 61–73. 17135495 10.1196/annals.1373.006

[28] Carrique-Mas JJ, Medley GF, Green LE (2008) Risks for bovine tuberculosis in British cattle farms restocked after the foot and mouth disease epidemic of 2001. Prev Vet Med 84: 85–93. 18164499 10.1016/j.prevetmed.2007.11.001

[29] Gopal R, Goodchild A, Hewinson G, de la Rua Domenech R, Clifton-Hadley R (2006) Introduction of bovine tuberculosis to north-east England by bought-in cattle. Vet Rec 159: 265–271. 16946308 10.1136/vr.159.9.265

[30] Whipple DL, Bolin CA, Miller JM (1996) Distribution of lesions in cattle infected with Mycobacterium bovis. J Vet Diagn Invest 8: 351–354. 8844579 10.1177/104063879600800312

[31] Thomson B (2006) Polymerase Chain Reaction detection of Mycobacteria tuberculosis complex in formalin fixed tissues. In: Thoen CO, Steele FH, Gilsdorf MJ, editors. Mycobacterium bovis Infection in Animals and Humans. 2^nd^ ed. Ames, Iowa, USA: Iowa State University Press. pp. 63–67.

[32] Leite CQ, Anno IS, Leite SR, Roxo E, Morlock GP, et al (2003) Isolation and identification of mycobacteria from livestock specimens and milk obtained in Brazil. Mem Inst Oswaldo Cruz 98: 319–323. 12886409 10.1590/s0074-02762003000300005

[33] Shitaye JE, Getahun B, Alemayehu T, Skoric M, Treml F, Fictum P, Vrbas V, Pavlik I (2006) A prevalence study of bovine tuberculosis by using abattoir meat inspection and tuberculin skin testing data, histopathological and IS6110 PCR examination of tissues with tuberculous lesions in cattle in Ethiopia. Veterinarni Medicina 51: 512–522.

[34] Tsegaye W, Aseffa A, Mache A, Mengistu Y, Berg S, et al (2010) Conventional and Molecular Epidemiology of Bovine Tuberculosis in Dairy Farms in Addis Ababa City, the Capital of Ethiopia. The International Journal of Applied Research in Veterinary Medicine 8: 143–151.

[35] Spicic S, Pate M, Duvnjak S, Katalinic-Jankovic V, Obrovac M, Dezdek D, Kompes G, Habrun B, Ocepek M, Cvetnic Z (2012) Molecular epidemiology of Mycobacterium tuberculosis transmission between cattle and man - a case report. Veterinarski Arhiv 82: 303–310.

[36] Mathews F, Macdonald DW, Taylor GM, Gelling M, Norman RA, et al (2006) Bovine tuberculosis (Mycobacterium bovis) in British farmland wildlife: the importance to agriculture. Proc Biol Sci 273: 357–365. 16543179 10.1098/rspb.2005.3298 PMC1560044

